# A new typical finding in late gadolinium enhanced images for the diagnosis of endomyocardial fibrosis - the double V sign

**DOI:** 10.1186/1532-429X-13-S1-O40

**Published:** 2011-02-02

**Authors:** Adriano C Carneiro, Roberta I Mochiduky, Leonardo F Zancaner, Estevan V Cabeda, Valeria M Moreira, Mario S Ribeiro, Alexandre V Villa, Roberto Kalil, Filho, Vera M Salemi, Charles Mady, Carlos E Rochitte

**Affiliations:** 1 Heart Institute -InCor- University of Sao Paulo Medical School, Sao Paulo, Brazil

## Introduction

Endomyocardial fibrosis (EMF) is a restrictive cardiomyopathy presenting with ventricular apical filling, possibly containing fibrotic tissue with thrombus and/or calcification. Late gadolinium enhancement (LGE) can detect apical fibrosis in EMF patients, as a hyper intense linear image usually in a letter “V” like shape pointing to the ventricular apex. Recently, we have observed a pattern of a double layer hyper and hypo intense in LGE images, also in a “V” like shape, possibly corresponding to fibrosis plus thrombus/calcification.

## Purpose

Our objective was to investigate the frequency of fibrosis (single V) and fibrosis associated to thrombus/calcification (double V) in a group of patients with EMF.

## Methods

We retrospectively studied 44 patients with confirmed EMF that had undergone CMR exam in a 1.5T scanner (GE Signa, CVi) during clinical investigation. CMR exam included cine-SSFP with standard parameters, and LGE images acquired 10min after 0.2mmol/kg of gadolinium-based contrast and with the parameters: TR 7.2ms, TE 3.2ms, matrix 256 x 192, flip angle 20° and inversion time (TI) 150 to 250ms, number of excitations 2 and acquisition in every heart beat (1 RR). We adjusted TI to null the signal from normal myocardium after contrast. Two observers independently classified LGE long-axis images from all patients in 4 categories: absence of fibrosis, single V fibrosis, double V fibrosis plus thrombus/calcification and fibrotic tissue without V shape.

## Results

**Figure 1:**
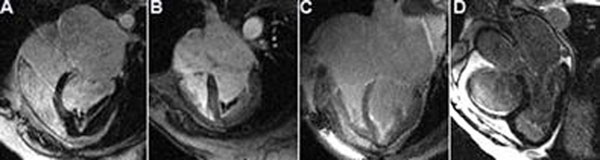


From the 44 patients, with mean age of 60 ± 11.8 years old, 36 (82%) were female. Thirty-nine patients (89%) presented apical fibrosis on the LGE images. From those, 21(54%) had the typical aspect of double-layered V shape of fibrosis plus thrombus/calcification, 11(28%) had only apical fibrosis (single V) and only 7 (18%) had fibrosis without the V shape (p<0.001 for the V sign, Figure 1: A/B, C and D, respectively).

From the 44 patients, 16 (36%) had a biventricular, 22 (50%) LV only, and 6 (14%) had RV only involvement by apical filling.

## Conclusion

In conclusion, more than half of patients (54%) with confirmed EMF and fibrosis presented an LGE imaging finding of apical filling associated to a hyper and hypo intense double-layered image, in a letter “V” like shape pointing to the ventricular apex (double V sign). Our data suggest that the double V sign can be considered a typical finding in EMF patients. This realization might help in the differential diagnosis with other apical filling conditions, such as apical hypertrophic cardiomyopathy, apical thrombus, and others.

